# Evaluation of Use of Epinephrine and Time to First Dose and Outcomes in Pediatric Patients With Out-of-Hospital Cardiac Arrest

**DOI:** 10.1001/jamanetworkopen.2023.5187

**Published:** 2023-03-28

**Authors:** Jeffrey Amoako, Sho Komukai, Junichi Izawa, Clifton W. Callaway, Masashi Okubo

**Affiliations:** 1 Department of Emergency Medicine, University of Maryland School of Medicine, Baltimore; 2 Division of Biomedical Statistics, Department of Integrated Medicine, Osaka University Graduate School of Medicine, Osaka, Japan; 3 Department of Internal Medicine, Okinawa Prefectural Chubu Hospital, Okinawa, Japan; 4 Department of Emergency Medicine, University of Pittsburgh School of Medicine, Pittsburgh, Pennsylvania

## Abstract

**Importance:**

While epinephrine has been widely used in prehospital resuscitation for pediatric patients with out-of-hospital cardiac arrest (OHCA), the benefit and optimal timing of epinephrine administration have not been fully investigated.

**Objectives:**

To evaluate the association between epinephrine administration and patient outcomes and to ascertain whether the timing of epinephrine administration was associated with patient outcomes after pediatric OHCA.

**Design, Setting, and Participants:**

This cohort study included pediatric patients (<18 years) with OHCA treated by emergency medical services (EMS) from April 2011 to June 2015. Eligible patients were identified from the Resuscitation Outcomes Consortium Epidemiologic Registry, a prospective OHCA registry at 10 sites in the US and Canada. Data analysis was performed from May 2021 to January 2023.

**Exposures:**

The main exposures were prehospital intravenous or intraosseous epinephrine administration and the interval between arrival of an advanced life support (ALS)–capable EMS clinician (ALS arrival) and the first administration of epinephrine.

**Main Outcomes and Measures:**

The primary outcome was survival to hospital discharge. Patients who received epinephrine at any given minute after ALS arrival were matched with patients who were at risk of receiving epinephrine within the same minute using time-dependent propensity scores calculated from patient demographics, arrest characteristics, and EMS interventions.

**Results:**

Of 1032 eligible individuals (median [IQR] age, 1 [0-10] years), 625 (60.6%) were male. 765 patients (74.1%) received epinephrine and 267 (25.9%) did not. The median (IQR) time interval between ALS arrival and epinephrine administration was 9 (6.2-12.1) minutes. In the propensity score–matched cohort (1432 patients), survival to hospital discharge was higher in the epinephrine group compared with the at-risk group (epinephrine: 45 of 716 [6.3%] vs at-risk: 29 of 716 [4.1%]; risk ratio, 2.09; 95% CI, 1.29-3.40). The timing of epinephrine administration was also not associated with survival to hospital discharge after ALS arrival (*P* for the interaction between epinephrine administration and time to matching = .34).

**Conclusions and Relevance:**

In this study of pediatric patients with OHCA in the US and Canada, epinephrine administration was associated with survival to hospital discharge, while timing of the administration was not associated with survival.

## Key Points

**Question:**

Is prehospital epinephrine administration associated with survival in pediatric patients with out-of-hospital cardiac arrest?

**Findings:**

In this cohort study with time-dependent propensity score and risk-set matching analyses of 1032 pediatric patients from a large out-of-hospital cardiac arrest registry in the US and Canada, prehospital epinephrine administration was associated with survival to hospital discharge.

**Meaning:**

These findings support use of epinephrine for pediatric out-of-hospital cardiac arrest.

## Teaser

This cohort study evaluates the association of epinephrine administration and time to first dose with survival in pediatric patients with out-of-hospital cardiac arrest.

## Introduction

Out-of-hospital cardiac arrest (OHCA) remains a public health challenge in the pediatric population. In the US, it is estimated that 7000 to 23 000 infants and children annually experience OHCA.^1,2^ The estimated rates of survival to hospital discharge and good functional recovery at hospital discharge after emergency medical services (EMS)-treated pediatric OHCA are 11.3% and 8.6%, respectively.^1^

Epinephrine is commonly administered at a dose of 0.01mg/kg of a 1:10 000 solution via intravenous and intraosseous routes during cardiopulmonary resuscitation to restore spontaneous circulation by augmenting coronary artery perfusion through the constriction of arterioles mediated by α-adrenergic effect and increasing aortic diastolic pressure.^3^ This pharmacological benefit might be more applicable for adults than children since cardiac arrest in children does not usually result from primary cardiac cause but is often due to progression of respiratory failure or shock. The 2020 American Heart Association Guidelines for Cardiopulmonary Resuscitation and Emergency Cardiovascular Care recommendations for administration of epinephrine continue to be reaffirmed, emphasizing early epinephrine administration.^3^ Though the guidelines suggest administering the initial dose of epinephrine for pediatric cardiac arrest within 5 minutes from the start of chest compression (level of evidence: C, limited data), evidence about the benefit and the optimal timing of epinephrine administration is extremely limited.^3,4,5^

The International Liaison Committee on Resuscitation (ILCOR) Pediatric Task Force published a systematic review about timing of epinephrine administration for pediatric cardiac arrest in 2021 and concluded that earlier administration of the first epinephrine dose could be more favorable in nonshockable pediatric cardiac arrest.^6^ However, all included studies had very serious overall risk of bias, and therefore, the results should be interpreted with caution. Importantly, none of the included studies in the systematic review addressed resuscitation time bias.^7,8^ In general, the longer resuscitation lasts, the more likely an intra-arrest intervention is to be performed.^7^ In addition, longer resuscitation duration is associated with lower chance of favorable patient outcomes.^9,10^ Without accounting for resuscitation time bias, these intra-arrest interventions would be biased toward harmful effects.^7^ One approach to address this bias is time-dependent propensity score (PS) and risk-set matching analyses,^11,12,13,14,15,16^ which, to our knowledge, have not been used to study epinephrine administration for pediatric OHCA in North America. Our primary objective was to evaluate the association between prehospital intravenous or intraosseous epinephrine administration and patient outcomes. The secondary objective was to ascertain whether the timing of epinephrine administration was associated with patient outcomes using this methodology.

## Methods

### Study Design and Setting

We used the Resuscitation Outcomes Consortium (ROC) Epidemiologic Registry-Cardiac Arrest, a prospective standardized data collection of consecutive patients with OHCA.^17,18^ The ROC is a clinical research network that studied the treatment and outcomes of patients with OHCA at 10 regional coordinating sites in the US and Canada.^17,18^ The data included patient demographics, arrest characteristics, layperson and EMS interventions, post-resuscitation management, and patient outcomes. As patient demographics, race and ethnicity were captured from health record or reported by a patient or family whenever possible or by EMS personnel. We included race and ethnicity given the association of patient race and ethnicity with patient outcomes. Additional details of the ROC are provided in the eMethods in Supplement 1. The publicly available, deidentified data were obtained from the National Heart, Lung, and Blood Institute Biologic Specimen and Data Repository Information Coordinating Center (https://biolincc.nhlbi.nih.gov/home/). The institutional review boards at the University of Pittsburgh and Osaka University deemed the study exempt from regulations related to human participant research because publicly available deidentified data were used. We followed the Strengthening the Reporting of Observational Studies in Epidemiology (STROBE) reporting guideline.

### Study Participants

**Figure 1.**
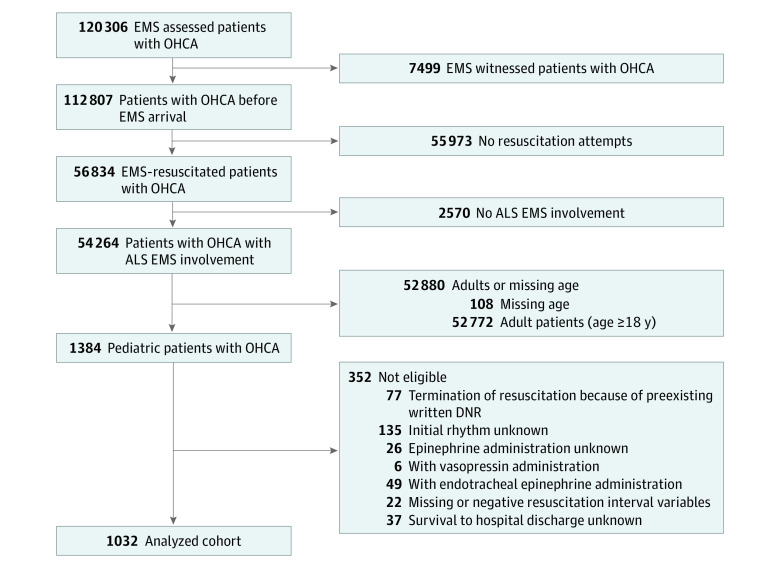
Flow Diagram of Cohort Selection ALS indicated advanced life support; DNR, do-not-resuscitate; EMS, emergency medical services; OHCA, out-of-hospital cardiac arrest.

We included pediatric patients (age <18 years) with EMS-treated, nontraumatic OHCA from April 2011 to June 2015, defined as resuscitation attempts with shock delivery by an external defibrillator (by layperson or EMS clinician) or chest compressions (by EMS clinician). We excluded patients who had termination of resuscitation (TOR) because of a preexisting do-not-resuscitate order, whose initial rhythm was unknown, whose epinephrine administration status was unknown, for whom vasopressin was administered, for whom epinephrine was administered via endotracheal tube, who had missing or negative resuscitation interval variables, and lastly, who had missing data about survival to hospital discharge (Figure 1). Resuscitation time variables included intervals between 911 call and first EMS vehicle arrival (EMS response time), between an advanced life support (ALS)–capable EMS clinician arrival (ALS arrival) and shock delivery by ALS clinician (if an ALS clinician delivered shock), between ALS arrival and the first epinephrine administration (if a patient received epinephrine), between ALS arrival and advanced airway management (AAM) (if a patient received AAM), between ALS arrival and departure from the scene (if a patient was transported), between ALS arrival and prehospital return of spontaneous circulation (ROSC) (if a patient had ROSC), between ALS arrival and prehospital TOR (if a patient had TOR), and between ALS arrival and hospital arrival (if a patient was transported).

### Exposure

The main exposures were prehospital intravenous (IV) or intraosseous (IO) epinephrine administration and the interval between ALS arrival and the first epinephrine administration. The interval was defined in whole minutes; epinephrine administration at 0 minutes indicated that the patient received epinephrine within the same minute of ALS arrival.

### Outcome Exposure

The primary outcome was survival to hospital discharge. Secondary outcomes included favorable functional outcome at hospital discharge, defined as modified Rankin scale score 3 or greater, and prehospital ROSC.

### Statistical Analysis

We reported patient demographics, cardiac arrest characteristics, and EMS interventions, stratified by patients who received and did not receive epinephrine. To address missing data for functional outcome, we conducted multiple imputation by chained equation, assuming missing at random.^19^ 20 imputed data sets were created through this process, which was carried out after risk-set matching described below. We rounded decimal places to use whole numbers when the number of imputed patients with favorable functional outcome had decimal points.

To evaluate the association between epinephrine administration and outcomes, we performed time-dependent PS and risk-set matching analyses.^11,12,13,14,15,20,21^ Since survival after OHCA differs between age groups of younger than 1 year and 1 year or older,^22^ we divided the whole cohort into 2 age groups, younger than 1 year and 1 year or older, and carried out the time-dependent PS and risk-set matching in each age group to avoid matching across age groups. We calculated PS as the time-varying probability of receiving epinephrine using a competing risk time-to-event analysis, Fine-Gray regression model.^13,14,15,16,23^ In the model, time to receiving the first epinephrine was the dependent variable, and ALS arrival was the time 0 because patients were at-risk of receiving epinephrine only after this time point. We included time-dependent and time-independent covariates shown in Table 1. Additional methodological details are provided in the eMethods in Supplement 1.

**Table 1.** Characteristics of Pediatric Patients With Out-of-Hospital Cardiac Arrest With and Without Epinephrine in Original Cohort

Characteristic			
	No epinephrine, No. (%) (n = 267)	Epinephrine, No. (%) (n = 765)	Standardized difference
Age, median (IQR), y	0 (0-3)	1 (0-11)	0.319
Age category, y			
<1	138 (51.7)	304 (39.7)	0.242
≥1	129 (48.3)	461 (60.3)	
Sex			
Female	106 (39.7)	301 (39.3)	0.007
Male	161 (60.3)	464 (60.7)	
Ethnicity			
Hispanic	21 (7.9)	82 (10.7)	0.152
Non-Hispanic	185 (69.3)	548 (71.6)	
Unknown	61 (22.8)	135 (17.6)	
Race			
Black	58 (21.7)	150 (19.6)	0.075
Multiple races	0	1 (0.1)	
White	47 (17.6)	133 (17.4)	
Other^a^	3 (1.1)	9 (1.2)	
Unknown	159 (59.6)	472 (61.7)	
Etiology			
Obvious cause	51 (19.1)	224 (29.3)	0.239
No obvious cause	216 (80.9)	541 (70.7)	
Initial rhythms			
Shockable rhythms	28 (10.5)	46 (6.0)	0.165
PEA	45 (16.9)	128 (16.7)	
Asystole	194 (72.7)	591 (77.3)	
Location			
Street/highway	3 (1.1)	15 (2.0)	0.213
Public building	11 (4.1)	16 (2.1)	
Place of recreation	8 (3.0)	32 (4.2)	
Home	226 (84.6)	660 (86.3)	
Healthcare facility	7 (2.6)	10 (1.3)	
Residential institution	1 (0.4)	7 (0.9)	
Other public property	9 (3.4)	20 (2.6)	
Other nonpublic property	2 (0.7)	3 (0.4)	
Unknown	0	2 (0.3)	
Witnessed collapse			
Bystander	68 (25.5)	156 (20.4)	0.132
Unwitnessed	191 (71.5)	577 (75.4)	
Unknown	8 (3.0)	32 (4.2)	
Layperson CPR			
Yes	170 (63.7)	466 (60.9)	0.057
No	88 (33.0)	272 (35.6)	
Unknown	9 (3.4)	27 (3.5)	
Shock delivery before ALS arrival			
Yes	8 (3.0)	14 (1.8)	0.096
No	259 (97.0)	751 (98.2)	
EMS response time [interval between 911 call and first EMS arrival], median (IQR), m	5.8 (4.6-8.0)	6.2 (4.7-8.9)	0.135
Shock delivery after ALS arrival			
Yes	20 (7.5)	67 (8.8)	0.046
No	247 (92.5)	698 (91.2)	
Advanced airway management			
Yes	58 (21.7)	431 (56.3)	0.759
No	209 (78.3)	334 (43.7)	
Departure from the scene			
Yes	233 (87.3)	612 (80.0)	0.197
No	34 (12.7)	153 (20.0)	
Interval between ALS arrival and epinephrine administration, median (IQR), minutes	NA	9.0 (6.2-12.1)	NA

Abbreviations: ALS, advanced life support; CPR, cardiopulmonary resuscitation; EMS, emergency medical services; NA, not applicable; PEA, pulseless electrical activity.

^a^ Other includes Asian, Native American, Pacific Islander, and other races.

To evaluate the association of epinephrine administration with outcomes, we performed 1:1 risk-set matching with replacement using the calculated time-dependent PS (See eMethods in Supplement 1 for the details).^7,12,13,14,15,16,20^ Each patient who received epinephrine at any given minute after ALS arrival was sequentially matched with a patient who was at risk of receiving epinephrine and had a similar PS at the same minute. These at-risk patients could have subsequently been administered epinephrine after the matching or never received epinephrine because matching should be independent of future events.^7,12,13,14,15,16,20,21^ At-risk patients could have been matched multiple times as at-risk patients or patients receiving epinephrine (only if the patients received epinephrine) until receiving epinephrine (matching with replacement).^12,13,16,24^ We set the caliper width for the nearest neighbor matching at 0.2 SD of the PS in the logit scale.^24,25^ To assess the performance of the risk-set matching, we calculated a standardized difference for each covariate. Standardized differences of less than 0.25 were regarded as a well-balanced match.^24^ We subsequently combined the matched cohort of each age group and created the whole matched cohort.

In the whole matched cohort, to assess the association between epinephrine administration and each outcome, we fitted a log link function in generalized estimating equations (GEEs) and estimated risk ratios (RRs) with 95% CIs.^26^ RRs represented the estimated magnitude of the association of epinephrine administration with outcomes, compared with that of those at risk of receiving epinephrine. GEEs were used to address potential within-pair correlation of risk set matching.^13,14,15,16^ We used frequency weighting adjustment because some patients in the at-risk group could not be independent because of the matching with replacement.^24^

To evaluate the timing of epinephrine administration, we fitted 2 models with log link function in GEEs with frequency weighting adjustment. One model treated the timing of the first epinephrine administration as a categorical variable by 5-minute intervals. The other model treated timing of the first epinephrine administration as a continuous variable. In the model with the continuous variable, we included an interaction term between the first epinephrine administration and time to matching (ie, time from ALS arrival to the time of matching) and estimated the RRs of epinephrine at each minute, assuming a linear relation between each outcome and the timing of epinephrine administration. When the *P* value for the interaction term was significant (*P* < .05), we considered the timing of epinephrine administration to be associated with the outcome.

In addition, we performed a sensitivity analysis. We excluded those who had ROSC or TOR within 5 minutes of ALS arrival on scene as these patients were successfully resuscitated or resuscitative efforts were terminated before epinephrine could have been feasibly administered. This analysis was also carried out using time-dependent PS and risk-set matching analyses with the same covariates, competing risks, and a censoring event. All tests were 2-sided, and we regarded *P* < .05 as statistically significant. All statistical analyses were performed with R software, version 3.5.1 (R Project for Statistical Computing). Data were analyzed from May 2021 to January 2023.

## Results

A total of 1032 pediatric patients were eligible in this analysis (Figure 1). Median (IQR) age was 1 (0-10) years, and 625 (60.6%) were male. Table 1 shows the patient demographics, cardiac arrest characteristics, and EMS interventions in the original cohort, stratified by patients who received and did not receive epinephrine. 765 patients received epinephrine (IV, 199 patients [26.0%]; IO, 552 patients [72.2%]; unable to determine IV or IO, 14 patients [1.8%]) and 267 patients did not receive epinephrine. The median (IQR) time interval between ALS arrival and epinephrine administration was 9 (6.2-12.1) minutes. Functional outcome data were missing in 60 patients (5.8%).

Using risk-set matching, 716 patients who received epinephrine (IV, 185 patients [25.8%]; IO, 519 patients [72.5%]; unable to determine IV or IO, 12 patients [1.7%]) were matched with patients who were at risk of receiving epinephrine in the same minutes (Table 2). Among those matched as at-risk patients, 483 patients (67.5%) received epinephrine after the matching. In this matched cohort, standardized differences were 0.138 or less for all variables, suggesting a good post-matching balance. The median (IQR) time interval between ALS arrival and administration of epinephrine was 8 (6-11) minutes for patients in the epinephrine group and 12 (9.5-16) minutes in the at-risk group.

**Table 2.** Characteristics of Pediatric Patients With Out-of-Hospital Cardiac Arrest With Epinephrine and at Risk of Receiving Epinephrine in Time-Dependent Propensity Score Matched Cohort

Characteristic	At risk of receiving epinephrine, No. (%) (n = 716)	Epinephrine, No. (%) (n = 716)	Standardized difference
Age, median (IQR), y	1 (0-10)	1 (0-11)	0.067
Age category, y			
<1	285 (39.8)	285 (39.8)	<0.001
≥1	431 (60.2)	431 (60.2)	
Sex			
Female	287 (40.1)	280 (39.1)	0.020
Male	429 (59.9)	436 (60.9)	
Ethnicity			
Hispanic	76 (10.6)	78 (10.9)	0.051
Non-Hispanic	503 (70.3)	515 (71.9)	
Unknown	137 (19.1)	123 (17.2)	
Race			
Black	143 (20.0)	142 (19.8)	0.022
Multiple races	1 (0.1)	1 (0.1)	
White	120 (16.8)	126 (17.6)	
Other^a^	8 (1.1)	8 (1.1)	
Unknown	444 (62.0)	439 (61.3)	
Etiology			
Obvious cause	221 (30.9)	206 (28.8)	0.046
No obvious cause	495 (69.1)	510 (71.2)	
Initial rhythms			
Shockable rhythms	28 (3.9)	42 (5.9)	0.102
PEA	108 (15.1)	118 (16.5)	
Asystole	580 (81.0)	556 (77.7)	
Location			
Street/highway	10 (1.4)	14 (2.0)	0.077
Public building	15 (2.1)	15 (2.0)	
Place of recreation	23 (3.2)	25 (3.5)	
Home	630 (88.0)	626 (87.4)	
Healthcare facility	7 (1.0)	6 (0.8)	
Residential institution	7 (1.0)	7 (1.0)	
Other public property	17 (2.4)	19 (2.7)	
Other nonpublic property	4 (0.6)	3 (0.4)	
Unknown	3 (0.4)	1 (0.1)	
Witnessed collapse			
Bystander	138 (19.3)	143 (20.0)	0.021
Unwitnessed	545 (76.1)	542 (75.7)	
Unknown	33 (4.6)	31 (4.3)	
Layperson CPR			
Yes	449 (62.7)	442 (61.7)	0.034
No	246 (34.4)	249 (34.8)	
Unknown	21 (2.9)	25 (3.5)	
Shock delivery before ALS arrival			
Yes	10 (1.4)	14 (2.0)	0.055
No	706 (98.6)	702 (98.0)	
EMS response time (interval between 911 call and first EMS arrival), median (IQR), minutes	6.0 (4.6-8.7)	6.2 (4.7-8.8)	0.025
Shock delivery after ALS arrival before matching			
Yes	16 (2.2)	30 (4.2)	0.111
No	700 (97.8)	686 (95.8)	
Advanced airway management before matching			
Yes	160 (22.3)	151 (21.1)	0.030
No	556 (77.7)	565 (78.9)	
Departure from the scene before matching			
Yes	141 (19.7)	104 (14.5)	0.138
No	575 (80.3)	612 (85.5)	
Epinephrine administration			
Yes	483 (67.5)	716 (100)	NA
Interval between ALS arrival and epinephrine administration, median (IQR), minutes	12 (9.5-16)	8 (6-11)	NA

Abbreviations: ALS, advanced life support; CPR, cardiopulmonary resuscitation; EMS, emergency medical services; NA, not applicable; PEA, pulseless electrical activity.

^a^ Other includes Asian, Native American, Pacific Islander, and other races.

In the primary analysis, receiving epinephrine was associated with survival to hospital discharge (6.3% vs 4.1%; RR, 2.09; 95% CI, 1.29-3.40) and prehospital ROSC (17.0% vs 11.9%; RR, 1.44; 95% CI 1.09-1.91), compared with those at risk of receiving epinephrine (Table 3). Epinephrine administration was not associated with favorable functional outcome at hospital discharge (4.9% vs 3.3%; RR, 1.61; 95% CI, 0.87-2.97).

**Table 3.** Outcomes in Time-Dependent Propensity Score Matched Cohort

Outcomes	Patients with outcome/total patients, No./No. (%)		Risk ratio (95% CI)
	At risk of receiving epinephrine	Epinephrine	
Primary analysis			
Survival to hospital discharge	29/716 (4.1)	45/716 (6.3)	2.09 (1.29-3.40)
Favorable functional outcome at hospital discharge	24/716 (3.3)	35/716 (4.9)	1.61 (0.87-2.97)
Prehospital ROSC	85/716 (11.9)	122/714 (17.0)	1.44 (1.09-1.91)
Sensitivity analysis (excluding those who had ROSC or TOR within 5 min after ALS arrival)			
Survival to hospital discharge	36/713 (5.0)	45/713 (6.3)	1.38 (0.87-2.19)
Favorable functional outcome at hospital discharge	27/713 (3.7)	32/713 (4.5)	1.23 (0.67-2.25)
Prehospital ROSC	87/713 (12.2)	123/713 (17.3)	1.48 (1.12-1.97)

Abbreviations: ALS, advanced life support; ROSC, return of spontaneous circulation; TOR, termination of resuscitation.

**Figure 2.**
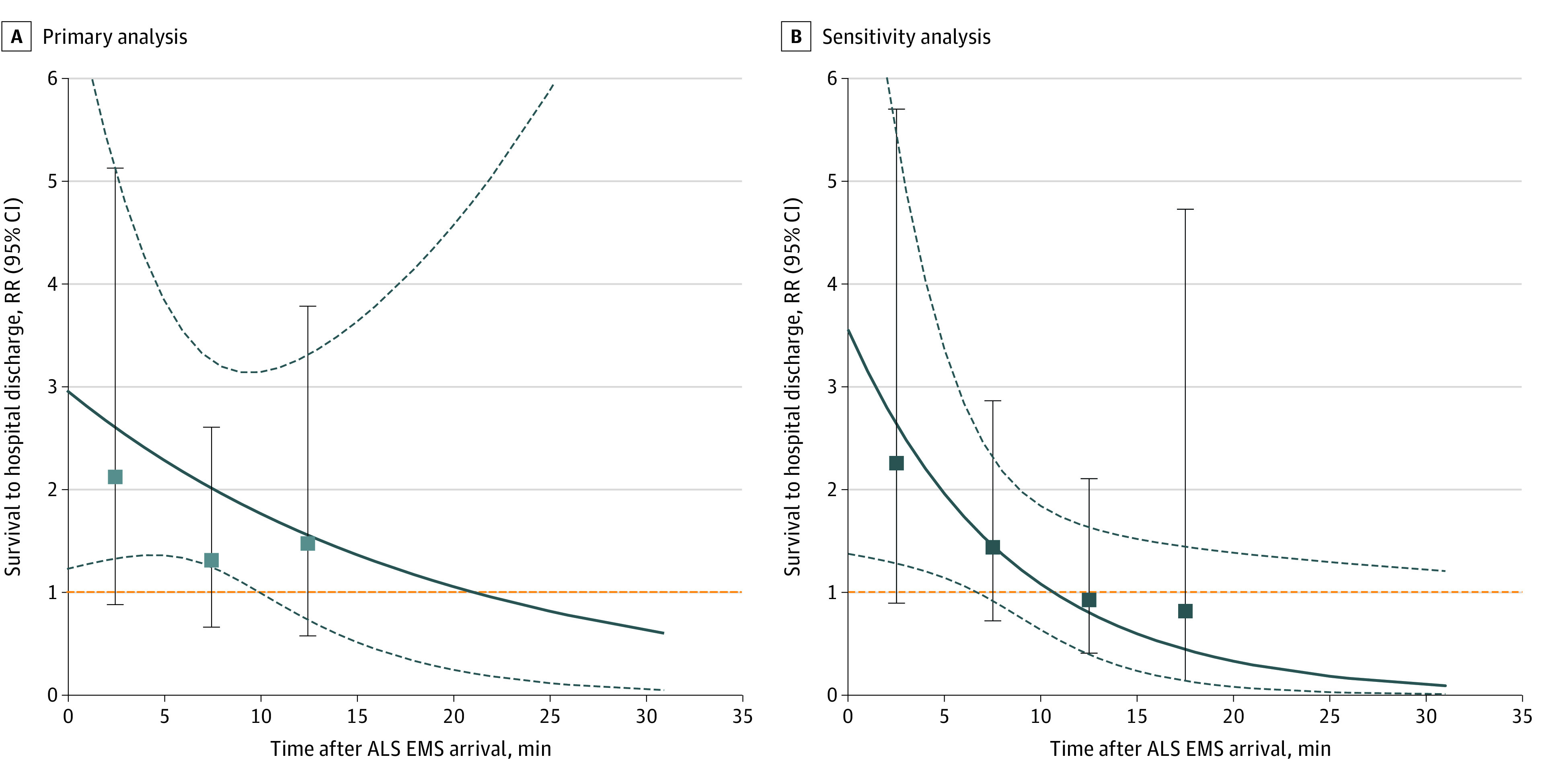
Survival to Hospital Discharge Stratified by Timing of Epinephrine Administration in Primary Analysis and Sensitivity Analysis (Excluding Those Who Had Return of Spontaneous Circulation or Termination of Resuscitation Within 5 Minutes of Advanced Life Support Arrival) Point estimates of the association of epinephrine with the outcome (solid lines) were reported with 95% CIs (dashed lines), treating timing of epinephrine administration after ALS arrival as a continuous variable. Squares indicate point estimates of the association of epinephrine with the outcome with 95% CIs, treating timing as a categorical variable. Figure 2A, *P* = .34 for the interaction between epinephrine administration and time to matching. Figure 2B, *P* = .03 for the interaction between epinephrine administration and time to matching. ALS indicates advanced life support; EMS emergency medical services.

Figure 2, eTable 1, and eFigures 1 and 2 in Supplement 1 show the RRs of epinephrine administration with outcomes stratified according to the timing of the administration. Treating the timing of epinephrine administration as a continuous variable, the interaction between epinephrine administration and time to matching was not significant (*P *for interaction = 0.34). The RRs for favorable functional outcome at hospital discharge (eFigure 1A and eTable 1 in Supplement 1) and prehospital ROSC (eFigure 2A and eTable 1 in Supplement 1) were shown. The interactions were not significant for favorable functional outcome and prehospital ROSC.

In the sensitivity analysis excluding those who had ROSC or TOR within 5 minutes of ALS arrival, 713 patients who received epinephrine were matched with patients who were at risk of receiving epinephrine in the same minutes. We presented the patient demographics, cardiac arrest characteristics, and EMS interventions in the original cohort (eTable 2 in Supplement 1) and in the matched cohort (eTable 3 in Supplement 1). All variables in the matched cohort showed good post-matching balance. The RRs of epinephrine administration on favorable functional outcome (RR, 1.23; 95% CI, 0.67-2.25) and prehospital ROSC (RR, 1.48; 95% CI, 1.12-1.97) were similar to the results of the primary analysis, but epinephrine administration was not associated with survival to hospital discharge (RR, 1.38; 95% CI, 0.87-2.19) (Table 3). RRs of epinephrine administration associated with outcomes stratified according to the timing of epinephrine administration were also similar to the results of the primary analysis except timing of epinephrine was associated with survival (*P* for interaction = .03) (Figure 2B; eFigures 1 and 2 in Supplement 1).

## Discussion

In this cohort study with time-dependent PS and risk-set matching analyses from a large OHCA registry in North America including 1032 pediatric patients, we observed that epinephrine administration was associated with survival to hospital discharge and prehospital ROSC, compared with those at risk of receiving epinephrine, whereas epinephrine was not associated with favorable functional outcome at hospital discharge. We also observed that the timing of epinephrine administration was not associated with survival to hospital discharge, favorable functional recovery, or prehospital ROSC.

### Comparison With Previous Studies

There are no clinical trials that compared epinephrine vs placebo for pediatric OHCA. A recent clinical trial comparing epinephrine vs placebo for adult OHCA showed that the epinephrine group had higher 30-day survival (odds ratio [OR], 1.39; 95% CI, 1.06-1.82) and survival to hospital admission (OR 3.59; 95% CI, 3.14-4.12), while epinephrine did not improve favorable neurological outcome at hospital discharge (OR, 1.18; 95% CI, 0.86-1.61).^27^ Our findings are consistent with the results of this adult OHCA trial. A recent observational study of a nationwide Japanese OHCA registry from 2007 to 2016 demonstrated that epinephrine administration was not associated with 1-month survival (RR, 1.13; 95% CI, 0.67-1.93) or 1-month survival with favorable neurologic outcome (RR, 1.56; 95% CI, 0.61-3.96), while epinephrine was associated with prehospital ROSC (RR, 3.17; 95% CI, 1.54-6.54) among 608 PS-matched pediatric patients (aged 8-17 years) compared with those at risk of receiving epinephrine using time-dependent PS and risk-set matching analyses.^14^ It is worth noting that the Japanese study used the same statistical method, and the participants were aged 8-17 years because EMS clinicians were legally permitted to administer epinephrine for patients aged 8 years and younger. Analyzing the largest sample size to date, we expanded previous knowledge to those who were younger than 8 years and to prehospital care in the US and Canada.

Regarding the timing of epinephrine administration for pediatric cardiac arrest, the ILCOR Pediatric Task Force conducted systematic review and meta-analyses for which the last search was performed on March 11, 2020.^6^ The systematic review identified 4 observational studies that evaluated the timing of epinephrine administration for pediatric OHCA.^6,28,29,30,31^ Across the included studies, the certainty of evidence was very low.^6^ The meta-analyses showed that time to the first epinephrine administration of less than 15 minutes was associated with survival to hospital discharge (RR, 2.49; 95% CI, 1.30-4.77) and 30-day survival (RR, 5.78; 95% CI, 2.82-11.86), but not associated with survival with good neurological outcome (RR, 3.94; 95% CI, 0.99-15.64), compared with time to the first epinephrine administration of greater than 15 minutes.^6,28,29,30,31^ None of the included studies in the systematic review and meta-analyses accounted for resuscitation time bias which might have led the difference in survival between results in the meta-analyses and our study.^7,8^

### Implications

First, our results would support epinephrine administration for pediatric OHCA and provide evidence to complement current resuscitation guidelines. It is worth noting that this current study included 1432 patients in the PS-matched cohort and may have been underpowered to detect significant difference in favorable functional outcome since a clinical trial that showed an effect of epinephrine on survival after adult OHCA was designed to enroll 8000 patients to detect a risk ratio of 1.25 on 30-day survival. Our study results may justify a well-powered future clinical trial to evaluate an effect of epinephrine for pediatric OHCA, although relatively low incidence of pediatric OHCA would be one of the difficulties of conducting such a trial. Additionally, since our study population is heterogenous, including broad range of age and diverse causes of arrest, there may be a subset of patients who have more benefit from epinephrine. Further investigation of such phenotypes would be important since a future trial could focus on those phenotypes with efficient sample size. Second, given the sample size limitation in pediatric OHCA research and expected challenges of conducting future trials, our findings support enhancing a collaborative research network across multiple regional and national OHCA data sets and further advancing our knowledge of intra-arrest interventions for pediatric patients using robust observational study designs.^32^

### Limitations

This study has limitations. First, epinephrine administration and time to the administration may be associated with quality of EMS performance. High-performing EMS systems could be adherent with current resuscitation guidelines and may administer epinephrine early. Since information about EMS systems was not available, we were unable to account for such clustering of patients within EMS systems. We were also unable to adjust for unmeasured confounders such as time from onset of arrest to epinephrine administration. Patients who received epinephrine tended to receive AAM. Prior studies reported that AAM was associated with worse patient outcomes after pediatric cardiac arrest.^12,33^ Although we accounted for AAM in the PS models, it is possible that we were unable to fully adjust for AAM and residual confounding might have existed. Second, it is possible that confounding by indication may have affected our results.^34^ For example, EMS clinicians might not have administered epinephrine if they expected that a patient would have early ROSC without epinephrine or have early TOR according to clinical judgement. We attempted to account for confounding by indication and carried out a sensitivity analysis excluding those who had ROSC or TOR within 5 minutes of ALS arrival on scene. However, confounding by indication may have still existed. Third, our main exposure variable was epinephrine administration. We did not account for total dose or interval of epinephrine administration since our primary objective was to evaluate the association between epinephrine administration and patient outcomes. Fourth, heterogenicity of the study population (eg, broad range of age and diverse etiology of arrest) might limit interpretation of the results. Additionally, the results may not be externally valid at other EMS systems since selected EMS systems were included in ROC according to adherence to performance metrics, ability to conduct trials, and interest in participating research.

## Conclusions

In this observational study of pediatric OHCA in North America, we observed that epinephrine administration was associated with survival to hospital discharge and prehospital ROSC, but not with favorable functional outcome at hospital discharge. We also observed that the timing of epinephrine administration was not associated with survival to hospital discharge, favorable functional outcome, or prehospital ROSC. Overall, these findings support the administration of epinephrine for pediatric OHCA.

## Notes

Supplement 1.
eMethods.
**eTable 1. **Outcomes in Time-Dependent Propensity Score Matched Cohort, Categorized by 5-Minute Interval After Advanced Life Support Emergency Medical Services Arrival **eTable 2. **Characteristics of Pediatric Patients With Out-of-Hospital Cardiac Arrest With and Without Epinephrine in Original Cohort Excluding Those Who Had ROSC or TOR Within 5 Minutes After ALS Arrival **eTable 3. **Characteristics of Pediatric Patients With Out-of-Hospital Cardiac Arrest With Epinephrine and at Risk of Receiving Epinephrine in Time-Dependent Propensity Score Matched Cohort Excluding Those Who Had ROSC or TOR Within 5 Minutes After ALS Arrival **eFigure 1. **Favorable Functional Outcome at Hospital Discharge Stratified by Timing of Epinephrine Administration in Primary Analysis (A) and Sensitivity Analysis (Excluding Those Who Had Return of Spontaneous Circulation or Termination of Resuscitation Within 5 Minutes of Advanced Life Support Capable Emergency Medical Services Clinician Arrival) (B) **eFigure 2. **Prehospital Return of Spontaneous Circulation Stratified by Timing of Epinephrine Administration in Primary Analysis (A) and Sensitivity Analysis (Excluding Those Who Had Return of Spontaneous Circulation or Termination of Resuscitation Within 5 Minutes of Advanced Life Support Capable Emergency Medical Services Clinician Arrival) (B)

Supplement 2.
Data Sharing Statement
