# Supplementary material for: Evaluation of Use of Epinephrine and Time to First Dose and Outcomes in Pediatric Patients With Out-of-Hospital Cardiac Arrest
Source: JAMA Netw Open. 2023 Mar 28;6(3):e235187. doi: 10.1001/jamanetworkopen.2023.5187 (PMC10051078; doi:10.1001/jamanetworkopen.2023.5187)
Supplement: Supplement 1. — eMethods. eTable 1. Outcomes in Time-Dependent Propensity Score Matched Cohort, Categorized by 5-Minute Interval After Advanced Life Support Emergency Medical Services Arrival eTable 2. Characteristics of Pediatric Patients With Out-of-Hospital Cardiac Arrest With and Without Epinephrine in Original Cohort Excluding Those Who Had ROSC or TOR Within 5 Minutes After ALS Arrival eTable 3. Characteristics of Pediatric Patients With Out-of-Hospital Cardiac Arrest With Epinephrine and at Risk of Receiving Epinephrine in Time-Dependent Propensity Score Matched Cohort Excluding Those Who Had ROSC or TOR Within 5 Minutes After ALS Arrival eFigure 1. Favorable Functional Outcome at Hospital Discharge Stratified by Timing of Epinephrine Administration in Primary Analysis (A) and Sensitivity Analysis (Excluding Those Who Had Return of Spontaneous Circulation or Termination of Resuscitation Within 5 Minutes of Advanced Life Support Capable Emergency Medical Services Clinician Arrival) (B) eFigure 2. Prehospital Return of Spontaneous Circulation Stratified by Timing of Epinephrine Administration in Primary Analysis (A) and Sensitivity Analysis (Excluding Those Who Had Return of Spontaneous Circulation or Termination of Resuscitation Within 5 Minutes of Advanced Life Support Capable Emergency Medical Services Clinician Arrival) (B) [file jamanetwopen-e235187-s001.pdf]

Supplemental Online Content

Amoako J, Komukai S, Izawa J, Callaway CW, Okubo M. Evaluation of use of epinephrine and time to first dose and outcomes in pediatric patients with out-of-hospital cardiac arrest. *JAMA Netw Open*. 2023;6(3):e235187.
doi:10.1001/jamanetworkopen.2023.5187

eMethods.
eTable 1. Outcomes in Time-Dependent Propensity Score Matched Cohort, Categorized by 5-Minute Interval After Advanced Life Support Emergency Medical Services Arrival
eTable 2. Characteristics of Pediatric Patients With Out-of-Hospital Cardiac Arrest With and Without Epinephrine in Original Cohort Excluding Those Who Had ROSC or TOR Within 5 Minutes After ALS Arrival
eTable 3. Characteristics of Pediatric Patients With Out-of-Hospital Cardiac Arrest With Epinephrine and at Risk of Receiving Epinephrine in Time-Dependent Propensity Score Matched Cohort Excluding Those Who Had ROSC or TOR Within 5 Minutes After ALS Arrival
eFigure 1. Favorable Functional Outcome at Hospital Discharge Stratified by Timing of Epinephrine Administration in Primary Analysis (A) and Sensitivity Analysis (Excluding Those Who Had Return of Spontaneous Circulation or Termination of Resuscitation Within 5 Minutes of Advanced Life Support Capable Emergency Medical Services Clinician Arrival) (B)
eFigure 2. Prehospital Return of Spontaneous Circulation Stratified by Timing of Epinephrine Administration in Primary Analysis (A) and Sensitivity Analysis (Excluding Those Who Had Return of Spontaneous Circulation or Termination of Resuscitation Within 5 Minutes of Advanced Life Support Capable Emergency Medical Services Clinician Arrival) (B)

This supplemental material has been provided by the authors to give readers additional information about their work.

© 2023 Amoako J et al. *JAMA Network Open.*

eMethods

Data Quality Control in the Resuscitation Outcomes Consortium (ROC)

The ROC used several quality assurance plans including periodic training of research teams in data collection and variable definitions, review of randomly selected records at each site by the data coordinating center, and logic checks of data element range and consistency in both the web-based data entry forms and the batch upload process.[1] Additionally, the data coordinating center conducted annual site visits to further review a portion of entered records, data capture process, and site-specific mechanisms for quality assurance.[1]

Time-dependent Propensity Score

We calculated propensity score as the time-varying probability of receiving epinephrine using a competing risk time-to-event analysis, Fine-Gray regression model.[2,3] In the model, time to receiving the first epinephrine was the dependent variable, and ALS arrival was the time 0. The time-dependent covariates were shock delivery (if a patient received shock), AAM (if a patient received AAM), and departure from the scene (if a patient was transported) after ALS arrival. The time-independent covariates were patient age, sex, ethnicity,[4] race,[4] etiology of arrest, initial rhythm, location of arrest, witness status, layperson CPR, shock delivery before ALS arrival, and EMS response time. We used spline functions (B-spline) for continuous variables (age and EMS response time). We chose these covariates a priori based on their association with survival from prior knowledge, biologic plausibility, and adequate ascertainment.[5-7] We included prehospital ROSC and TOR before the first dose of epinephrine administration

© 2023 Amoako J et al. *JAMA Network Open.*

as competing risks in the model because (1) epinephrine administration never occurred after ROSC or TOR except cases with re-arrest after ROSC, (2) our interest was epinephrine administration for the initial arrest, and (3) ROSC and TOR were informative censoring events; i.e., ROSC was strongly associated with patient outcomes, and TOR determined patient outcomes, which indicated that it was inaccurate to treat ROSC and TOR as noninformative censoring events.[3] We modelled hospital arrival as a censoring event because our interest was prehospital epinephrine administration.[3]

Risk-set Matching with Replacement

We used matching with replacement for two reasons: 1. Matching with replacement can often decrease bias because controls that are similar to many treated patients can be used multiple times, which is particularly helpful in settings where there are few controls and 2. The order in which the treated patients are matched does not matter—without replacement, the patients who had epinephrine later would not be able to find appropriate controls.[8]

References:

1. Morrison LJ, Nichol G, Rea TD, et al. Rationale, development and implementation of the Resuscitation Outcomes Consortium Epistry-Cardiac Arrest. *Resuscitation.* 2008;78(2):161-169.
2. Beyersmann J, Schumacher M. Time-dependent covariates in the proportional subdistribution hazards model for competing risks. *Biostatistics.* 2008;9(4):765-776.
3. Izawa J, Komukai S, Gibo K, et al. Pre-hospital advanced airway management for adults with out-of-hospital cardiac arrest: nationwide cohort study. *BMJ.* 2019;364:l430.
4. Blewer AL, Schmicker RS, Morrison LJ, et al. Variation in bystander cardiopulmonary resuscitation delivery and subsequent survival from out-of-

© 2023 Amoako J et al. *JAMA Network Open.*

hospital cardiac arrest based on neighborhood-level ethnic characteristics. *Circulation* 2020;141:34-41.
5. Topjian AA, Raymond TT, Atkins D, et al. Part 4: Pediatric Basic and Advanced Life Support: 2020 American Heart Association Guidelines for Cardiopulmonary Resuscitation and Emergency Cardiovascular Care. *Circulation.* 2020;142(16_suppl_2):S469-S523.
6. de Caen AR, Maconochie IK, Aickin R, et al. Part 6: Pediatric Basic Life Support and Pediatric Advanced Life Support: 2015 International Consensus on Cardiopulmonary Resuscitation and Emergency Cardiovascular Care Science With Treatment Recommendations. *Circulation.* 2015;132(16 Suppl 1):S177-203.
7. Maconochie IK, Aickin R, Hazinski MF, et al. Pediatric Life Support: 2020 International Consensus on Cardiopulmonary Resuscitation and Emergency Cardiovascular Care Science With Treatment Recommendations. *Circulation.* 2020;142(16_suppl_1):S140-S184.
8. Stuart EA. Matching methods for causal inference: A review and a look forward. *Stat Sci.* 2010;25(1):1-21.

© 2023 Amoako J et al. *JAMA Network Open.*

eTable 1. Outcomes in Time-Dependent Propensity Score Matched Cohort, Categorized by 5-Minute Interval After Advanced Life Support Emergency Medical Services Arrival

	No (%) patients with outcome/total patients		
Outcomes	At-risk of receiving epinephrine	Epinephrine	Risk ratio (95% CI)
**Primary analysis**			
Survival to hospital discharge, minutes after ALS EMS arrival			
0 to <5	7/99 (7.1%)	13/99 (13.1%)	2.12 (0.88-5.13)
5 to <10	14/337 (4.2%)	20/337 (5.9%)	1.31 (0.66-2.60)
10 to <15	8/212 (3.8%)	10/212 (4.7%)	1.47 (0.57-3.78)
15 to <20	0/53 (0%)	2/53 (3.8%)	*
≥20	0/15 (0%)	0/15 (0%)	*
Favorable functional outcome at hospital discharge, minutes after ALS EMS arrival			
0 to <5	6/99 (6.1%)	10/99 (10.1%)	2.58 (0.71-9.37)
5 to <10	13/337 (3.9%)	16/337 (4.7%)	1.35 (0.57-3.20)
10 to <15	6/212 (2.8%)	8/212 (3.8%)	2.32 (0.41-12.95)
15 to <20	0/53 (0%)	1/53 (1.9%)	*
≥20	0/15 (0%)	0/15 (0%)	*
Prehospital ROSC, minutes after ALS EMS arrival			
0 to <5	16/99 (16.2%)	36/99 (36.4%)	2.28 (1.33-3.90)
5 to <10	48/337 (14.2%)	53/337 (15.7%)	1.05 (0.72-1.53)
10 to <15	18/212 (8.5%)	24/212 (11.3%)	1.26 (0.69-2.30)
15 to <20	2/53 (3.8%)	5/53 (9.4%)	3.07 (0.61-15.32)
≥20	1/15 (6.7%)	4/15 (26.7%)	6.13 (0.74-50.57)
**Sensitivity analysis**			

© 2023 Amoako J et al. *JAMA Network Open.*

Survival to hospital discharge, minutes after ALS EMS arrival			
0 to <5	6/98 (6.1%)	13/98 (13.3%)	2.26 (0.89-5.70)
5 to <10	14/335 (4.2%)	20/335 (6.0%)	1.44 (0.72-2.86)
10 to <15	13/213 (6.1%)	10/213 (4.7%)	0.92 (0.41-2.10)
15 to <20	3/54 (5.6%)	2/54 (3.7%)	0.81 (0.14-4.73)
≥20	0/13 (0%)	0/13 (0%)	*
Favorable functional outcome at hospital discharge, minutes after ALS EMS arrival			
0 to <5	5/98 (5.1%)	9/98 (9.2%)	2.40 (0.68-8.47)
5 to <10	12/335 (3.6%)	15/335 (4.5%)	1.22 (0.51-2.89)
10 to <15	8/213 (3.7%)	7/213 (3.3%)	1.21 (0.37-3.98)
15 to <20	1/54 (1.9%)	1/54 (1.9%)	*
≥20	0/13 (0%)	0/13 (0%)	*
Prehospital ROSC, minutes after ALS EMS arrival			
0 to <5	24/98 (24.5%)	36/98 (36.7%)	1.44 (0.93-2.23)
5 to <10	37/335 (11.0%)	53/335 (15.8%)	1.55 (1.03-2.34)
10 to <15	24/213 (11.3%)	24/213 (11.3%)	0.83 (0.48-1.45)
15 to <20	2/54 (3.7%)	6/54 (11.1%)	3.67 (0.76-17.65)
≥20	0/13 (0%)	4/13 (30.8%)	*

ALS indicates advanced life support; ROSC, return of spontaneous circulation; and TOR, termination of resuscitation.

* The model did not converge.

© 2023 Amoako J et al. *JAMA Network Open.*

eTable 2. Characteristics of Pediatric Patients With Out-of-Hospital Cardiac Arrest With and Without Epinephrine in Original Cohort Excluding Those Who Had ROSC or TOR Within 5 Minutes After ALS Arrival

	No epinephrine (n=240)	Epinephrine (n=765)	Standardized difference
Age, median (IQR), y	0 (0-3)	1 (0-11)	0.427
Age category, n (%)			0.292
<1 year	130 (54.2)	304 (39.7)	
≥1 year	110 (45.8)	461 (60.3)	
Sex, n (%)			0.039
Female	99 (41.2)	301 (39.3)	
Male	141 (58.8)	464 (60.7)	
Ethnicity			0.175
Hispanic	18 (7.5)	82 (10.7)	
Non-Hispanic	165 (68.8)	548 (71.6)	
Unknown	57 (23.8)	135 (17.6)	
Race, n (%)			0.091
Black	52 (21.7)	150 (19.6)	
Multiple races	0 (0)	1 (0.1)	
White	45 (18.8)	133 (17.4)	
Other[a]	2 (0.8)	9 (1.2)	
Unknown	141 (58.8)	472 (61.7)	
Etiology, n (%)			0.270
Obvious cause	43 (17.9)	224 (29.3)	
No obvious cause	197 (82.1)	541 (70.7)	
Initial rhythms, n (%)			0.080
Shockable rhythms	19 (7.9)	46 (6.0)	
PEA	37 (15.4)	128 (16.7)	

© 2023 Amoako J et al. *JAMA Network Open.*

Asystole	184 (76.7)	591 (77.3)	
Location, n (%)			0.202
Street/highway	2 (0.8)	15 (2.0)	
Public building	7 (2.9)	16 (2.1)	
Place of recreation	6 (2.5)	32 (4.2)	
Home	209 (87.1)	660 (86.3)	
Healthcare facility	6 (2.5)	10 (1.3)	
Residential institution	1 (0.4)	7 (0.9)	
Other public property	7 (2.9)	20 (2.6)	
Other non-public property	2 (0.8)	3 (0.4)	
Unknown	0 (0.0)	2 (0.3)	
Witnessed collapse, n (%)			0.072
Bystander	55 (22.9)	156 (20.4)	
Unwitnessed	177 (73.8)	577 (75.4)	
Unknown	8 (3.3)	32 (4.2)	
Layperson CPR, n (%)			0.033
Yes	150 (62.5)	466 (60.9)	
No	82 (34.2)	272 (35.6)	
Unknown	8 (3.3)	27 (3.5)	
Shock delivery before ALS arrival, n (%)			0.087
Yes	4 (1.7)	14 (1.8)	
No	236 (98.3)	751 (98.2)	
EMS response time (interval between 9-1-1 call and first EMS arrival), median (IQR), minutes	5.8 (4.7-8.0)	6.2 (4.7-8.9)	0.149
Shock delivery after ALS arrival, n (%)			0.078
Yes	16 (6.7)	67 (8.8)	
No	224 (93.3)	698 (91.2)	
Advanced airway management, n (%)			0.807
Yes	48 (20.0)	431 (56.3)	

© 2023 Amoako J et al. *JAMA Network Open.*

No	192 (80.0)	334 (43.7)	
Departure from the scene, n (%)			0.256
Yes	214 (89.2)	612 (80.0)	
No	26 (10.8)	153 (20.0)	
Interval between ALS arrival and epinephrine administration, median (IQR), minutes	N/A	9.0 (6.2-12.1)	N/A

AAM indicates advanced airway management; ALS, advanced life support; CPR, cardiopulmonary resuscitation; EMS, emergency medical services; IQR indicates interquartile range; N/A, not applicable; PEA, pulseless electrical activity; ROSC, return of spontaneous circulation; and TOR, termination of resuscitation.

[a] Other includes Asian, Native American, Pacific Islander, and other races.

© 2023 Amoako J et al. *JAMA Network Open.*

eTable 3. Characteristics of Pediatric Patients With Out-of-Hospital Cardiac Arrest With Epinephrine and At-Risk of Receiving Epinephrine in Time-Dependent Propensity Score Matched Cohort Excluding Those Who Had ROSC or TOR Within 5 Minutes After ALS Arrival

	At-risk of receiving epinephrine (n=713)	Epinephrine (n=713)	Standardized difference
Age, median (IQR), y	1 (0-11)	1 (0-11)	0.017
Age category, n (%)			<0.001
<1 year	283 (39.7)	283 (39.7)	
≥1 year	430 (60.3)	430 (60.3)	
Sex, n (%)			0.058
Female	262 (36.7)	282 (39.6)	
Male	451 (63.3)	431 (60.4)	
Ethnicity			0.030
Hispanic	77 (10.8)	77 (10.8)	
Non-Hispanic	505 (70.8)	513 (71.9)	
Unknown	131 (18.4)	123 (17.3)	
Race, n (%)			0.069
Black	148 (20.8)	143 (20.1)	
Multiple races	3 (0.4)	1 (0.1)	
White	117 (16.4)	126 (17.7)	
Other[a]	8 (1.1)	6 (0.8)	
Unknown	437 (61.3)	437 (61.3)	
Etiology, n (%)			0.095
Obvious cause	230 (32.3)	199 (27.9)	
No obvious cause	483 (67.7)	514 (72.1)	
Initial rhythms, n (%)			0.050
Shockable rhythms	46 (6.5)	42 (5.9)	
PEA	106 (14.9)	118 (16.5)	
Asystole	561 (78.7)	553 (77.6)	

© 2023 Amoako J et al. *JAMA Network Open.*

Location, n (%)			0.142
Street/highway	18 (2.5)	15 (2.1)	
Public building	13 (1.8)	15 (2.1)	
Place of recreation	35 (4.9)	25 (3.5)	
Home	595 (83.5)	620 (87.0)	
Healthcare facility	10 (1.4)	7 (1.0)	
Residential institution	5 (0.7)	7 (1.0)	
Other public property	31 (4.3)	19 (2.7)	
Other non-public property	5 (0.7)	3 (0.4)	
Unknown	1 (0.1)	2 (0.3)	
Witnessed collapse, n (%)			0.039
Bystander	143 (20.1)	146 (20.5)	
Unwitnessed	532 (74.6)	535 (75.0)	
Unknown	38 (5.3)	32 (4.5)	
Layperson CPR, n (%)			0.041
Yes	425 (59.6)	438 (61.4)	
No	263 (36.9)	249 (34.9)	
Unknown	25 (3.5)	26 (3.6)	
Shock delivery before ALS arrival, n (%)			0.071
Yes	15 (2.1)	14 (2.0)	
No	698 (97.9)	699 (98.0)	
EMS response time (interval between 9-1-1 call and first EMS arrival), median (IQR), minutes	6.2 (4.6-9.4)	6.2 (4.7-8.8)	0.040
Shock delivery after ALS arrival before matching, n (%)			0.052
Yes	23 (3.2)	30 (4.2)	
No	690 (96.8)	683 (95.8)	
Advanced airway management before matching, n (%)			0.024
Yes	143 (20.1)	150 (21.0)	
No	570 (79.9)	563 (79.0)	

© 2023 Amoako J et al. *JAMA Network Open.*

Departure from the scene before matching, n (%)			0.084
Yes	126 (17.7)	104 (14.6)	
No	587 (82.3)	609 (85.4)	
Epinephrine administration			
Yes, n (%)	473 (66.3)	713 (100)	N/A
Interval between ALS arrival and epinephrine administration, median (IQR), minutes	12 (9-16)	8 (6-11)	N/A

AAM indicates advanced airway management; ALS, advanced life support; CPR, cardiopulmonary resuscitation; EMS, emergency medical services; IQR indicates interquartile range; N/A, not applicable; PEA, pulseless electrical activity; ROSC, return of spontaneous circulation; and TOR, termination of resuscitation

[a] Other includes Asian, Native American, Pacific Islander, and other races.

© 2023 Amoako J et al. *JAMA Network Open.*

eFigure 1. Favorable Functional Outcome at Hospital Discharge Stratified by Timing of Epinephrine Administration in Primary Analysis (A) and Sensitivity Analysis (Excluding Those Who Had Return of Spontaneous Circulation or Termination of Resuscitation Within 5 Minutes of Advanced Life Support Capable Emergency Medical Services Provider Arrival) (B)

Point estimates of the association of epinephrine with the outcome (solid lines) were reported with 95% CIs (dashed lines), treating timing of epinephrine administration after ALS arrival as a continuous variable. Squares indicate point estimates of the association of epinephrine with the outcome with 95% CIs, treating timing as a categorical variable. We rounded decimal places to use whole numbers when imputing the number of patients with favorable functional outcome.

ALS indicates advanced life support; CI confidence interval; EMS emergency medical services.

© 2023 Amoako J et al. *JAMA Network Open.*

Supplemental Figure 1A

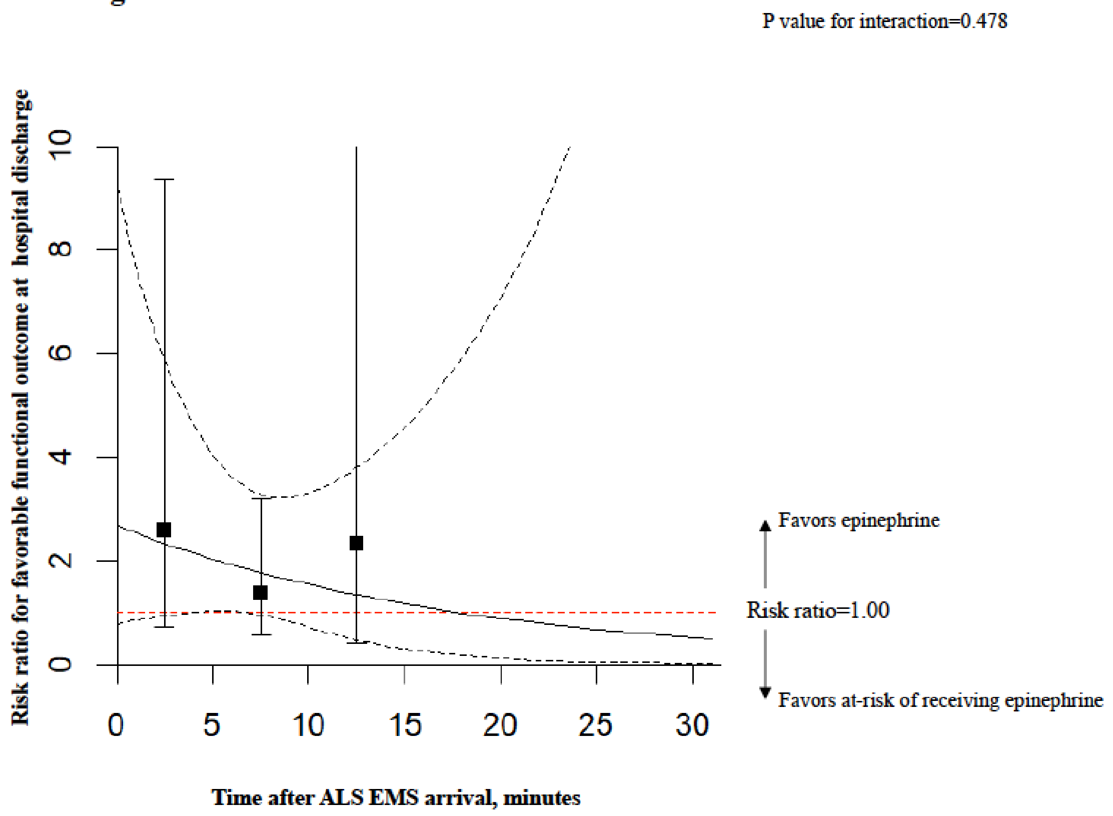

© 2023 Amoako J et al. *JAMA Network Open.*

Supplemental Figure 1B

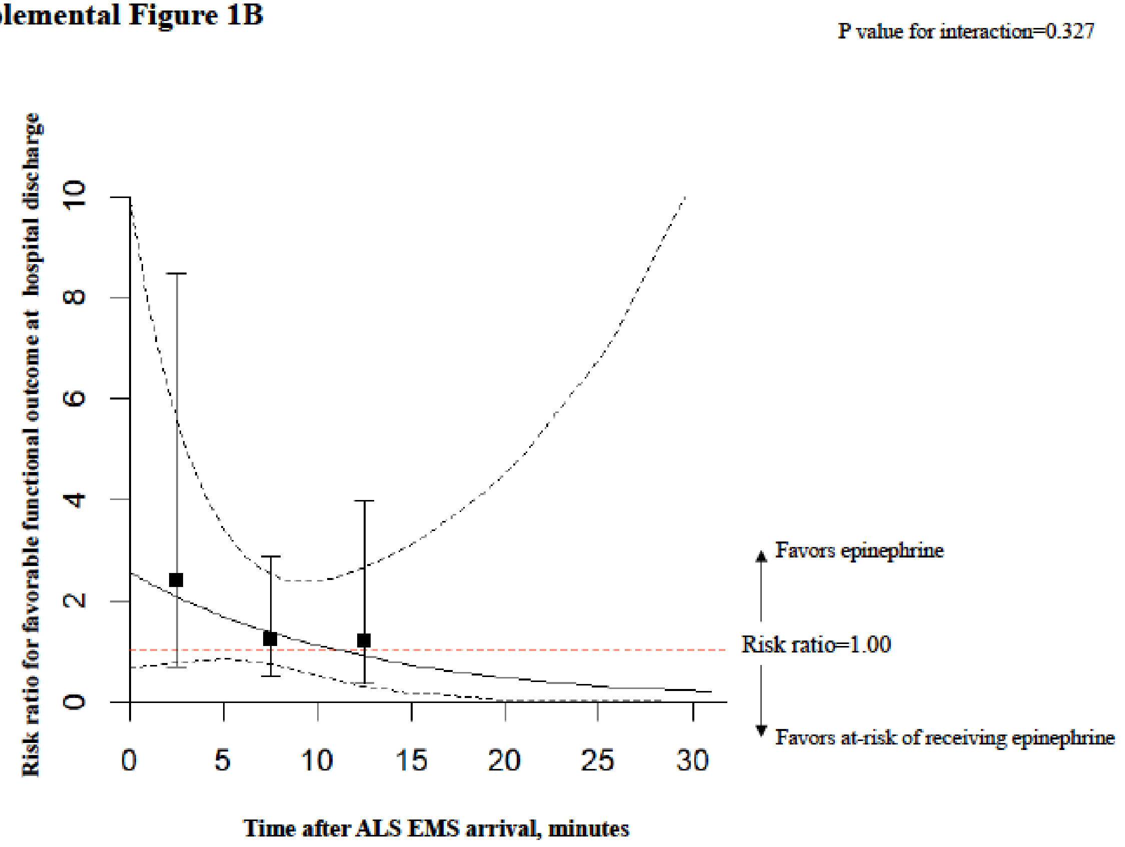

© 2023 Amoako J et al. *JAMA Network Open.*

eFigure 2. Prehospital Return of Spontaneous Circulation Stratified by Timing of Epinephrine Administration in Primary Analysis (A) and Sensitivity Analysis (Excluding Those Who Had Return of Spontaneous Circulation or Termination of Resuscitation Within 5 Minutes of Advanced Life Support Capable Emergency Medical Services Provider Arrival) (B)

Point estimates of the association of epinephrine with the outcome (solid lines) were reported with 95% CIs (dashed lines), treating timing of epinephrine administration after ALS arrival as a continuous variable. Squares indicate point estimates of the association of epinephrine with the outcome with 95% CIs, treating timing as a categorical variable.

ALS indicates advanced life support; CI confidence interval; EMS emergency medical services.

Supplemental Figure 2A

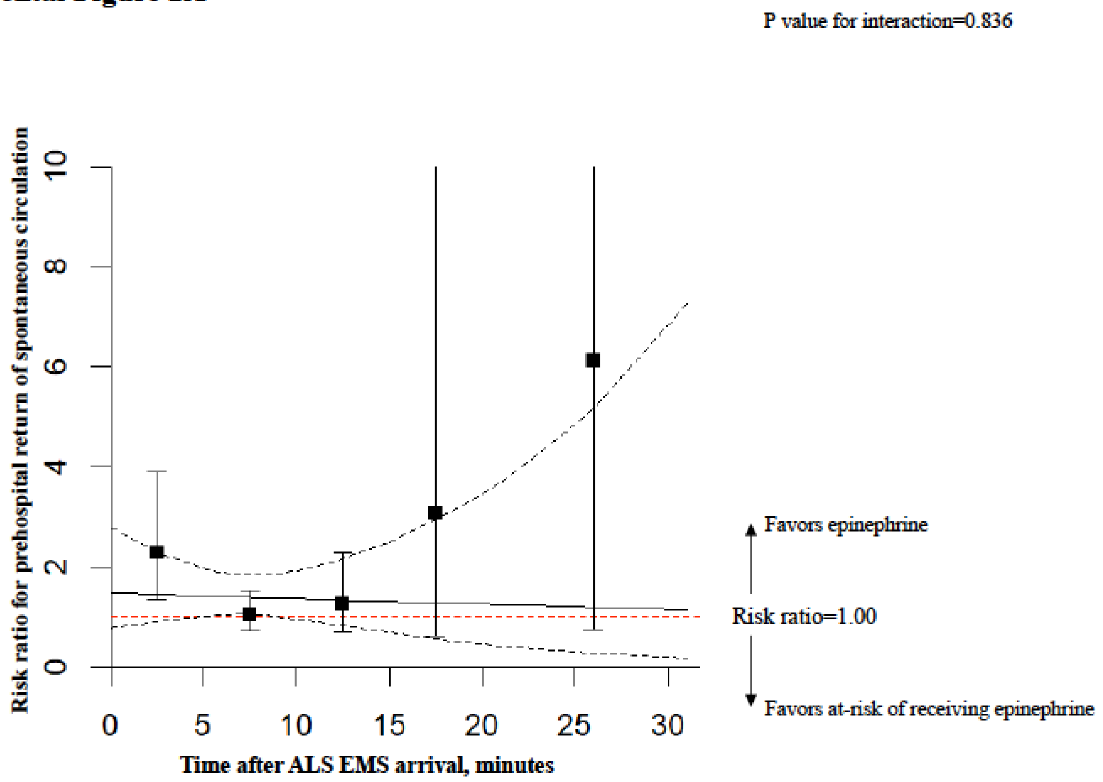

© 2023 Amoako J et al. *JAMA Network Open.*

Supplemental Figure 2B

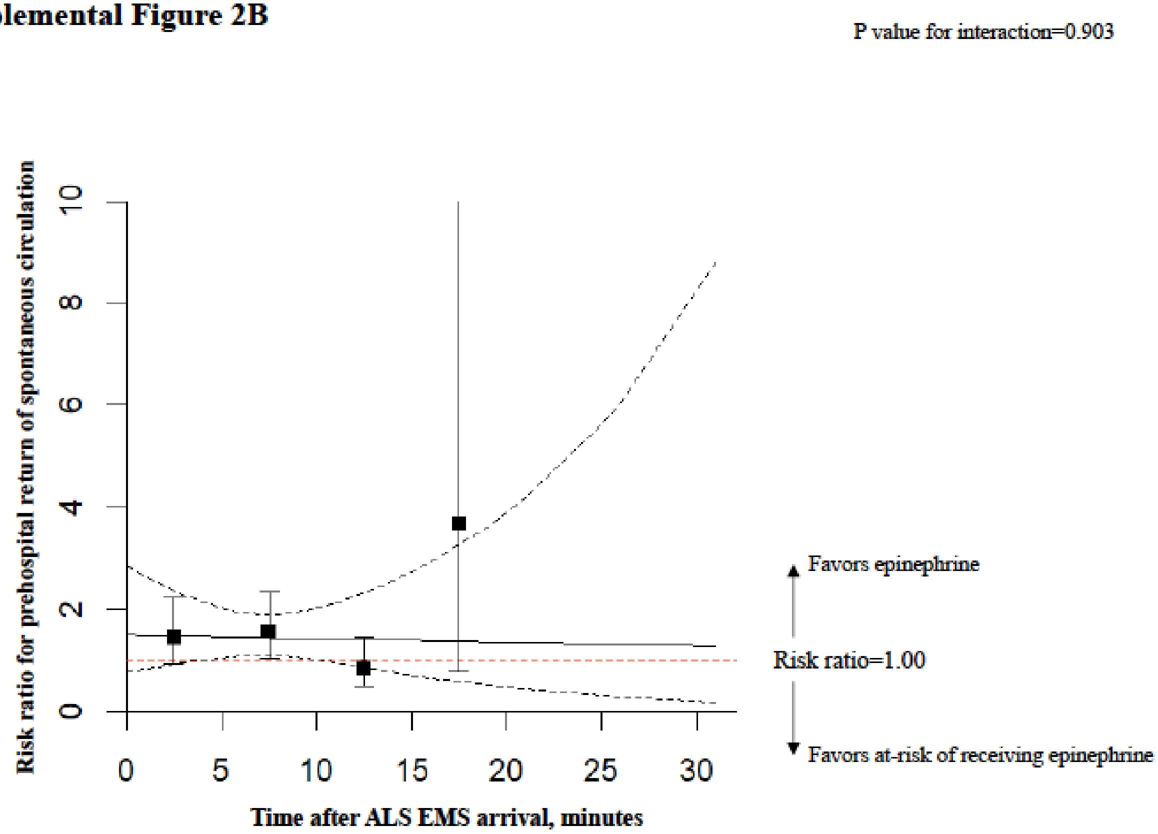

© 2023 Amoako J et al. *JAMA Network Open.*
