# Supplementary material for: Evaluation of Use of Epinephrine and Time to First Dose and Outcomes in Pediatric Patients With Out-of-Hospital Cardiac Arrest
Source: JAMA Netw Open. 2023 Mar 28;6(3):e235187. doi: 10.1001/jamanetworkopen.2023.5187 (PMC10051078; doi:10.1001/jamanetworkopen.2023.5187)
Supplement: Supplement 2. — Data Sharing Statement [file jamanetwopen-e235187-s002.pdf]

Data Sharing Statement

Amoako. Evaluation of Use of Epinephrine and Time to First Dose and Outcomes in Pediatric Patients With Out-of-Hospital Cardiac Arrest. *JAMA Netw Open*. Published March 28, 2023. doi:10.1001/jamanetworkopen.2023.5187

Data
Data available: Yes
Data types: Deidentified participant data
How to access data: https://biolincc.nhlbi.nih.gov/home/
When available: With publication

Supporting Documents
Document types: None

Additional Information
Who can access the data: Anyone can request the data from the NHLBI using the link above.
Types of analyses: Per National Heart, Lung and Blood Institute (NHLBI) Biologic Specimen and Data Repository Information Coordinating Center.
Mechanisms of data availability: National Heart, Lung and Blood Institute (NHLBI) Biologic Specimen and Data Repository Information Coordinating Center will review the application.
